# Supplementary material for: Content-rich biological network constructed by mining PubMed abstracts
Source: BMC Bioinformatics. 2004 Oct 8;5:147. doi: 10.1186/1471-2105-5-147 (PMC528731; doi:10.1186/1471-2105-5-147)
Supplement: Additional File 2 — The original results of the above study (non-essential files are deleted to keep the file size under the limit set by BMC bioinformatics). [file 1471-2105-5-147-S2.bz2 › chilibotAdditionalFile2/dip05/505ID10880444E10672/html/ACVR2_BMP2.html]

 


 **ACVR2** and **BMP2** 
  
Found 20 abstracts in PubMed, retrieved 05.  
 

 What does Google say? 
 PDF only 
| .edu only 

---

**Interactive relationship** (e.g. stimulation, inhibition, etc)

**Neutral relationship**- Activin receptor  [ **ACVR2** ]  like kinase ALK 1, ALK 2, ALK 3, and ALK 6 were able to reproduce the hypertrophic maturation of chondrocytes induced by BMP 2  [ **BMP2** ] .  Ref: 12082094 J Biol Chem, 2002
